# Link between cognitive neuroscience and education: the case of clinical assessment of developmental dyscalculia

**DOI:** 10.3389/fnhum.2015.00304

**Published:** 2015-05-28

**Authors:** Orly Rubinsten

**Affiliations:** Department of Learning Disabilities, Edmond J. Safra Brain Research Center for the Study of Learning Disabilities, University of HaifaHaifa, Israel

**Keywords:** neurocognitive processes, clinical assessments, numerical knowledge, dyscalculia, implicit processes

## Abstract

In recent years, cognitive neuroscience research has identified several biological and cognitive features of number processing deficits that may now make it possible to diagnose mental or educational impairments in arithmetic, even earlier and more precisely than is possible using traditional assessment tools. We provide two sets of recommendations for improving cognitive assessment tools, using the important case of mathematics as an example. (1) neurocognitive tests would benefit substantially from incorporating assessments (based on findings from cognitive neuroscience) that entail systematic manipulation of fundamental aspects of number processing. Tests that focus on evaluating networks of core neurocognitive deficits have considerable potential to lead to more precise diagnosis and to provide the basis for designing specific intervention programs tailored to the deficits exhibited by the individual child. (2) implicit knowledge, derived from inspection of variables that are irrelevant to the task at hand, can also provide a useful assessment tool. Implicit knowledge is powerful and plays an important role in human development, especially in cases of psychiatric or neurological deficiencies (such as math learning disabilities or math anxiety).

Data from evidence based teaching methods and cognitive neuroscience may provide the basis for development of more efficient and precise assessment and intervention tools for cases of learning disabilities (e.g., Sigman et al., 2014). The current review critically examines the need to develop cognitive assessment tools that are based on findings from research in cognitive neuroscience that highlight individual differences among learners. We use the very important case of mathematics and show the value of basing learning disability assessment tools on: (1) findings from cognitive neuroscience; and (2) the inspection of non-intentional cognitive variables.

## The Link Between Elemental Number Processing and Mathematical Abilities

Human symbolic arithmetical and higher mathematical abilities are unique phenomena, posing a challenge for the disciplines of the neurocognitive sciences and education. Mathematics provides an important intersection in the new synthesis between education, cognitive neuroscience, biology, and psychology, because it is an important entry point to knowledge required in our technological age. For example, math abilities have been found to predict academic achievements even better than reading (e.g., Estrada et al., 2004). However, many people find learning arithmetic or mathematics challenging (Dowker, 2005). Specifically, approximately 20% of people have low numeracy skills, a condition called developmental dyscalculia (DD; Rubinsten and Henik, 2009; Kaufmann et al., 2013; Szücs and Goswami, 2013), or mathematical learning disability (MLD; Gross-Tsur et al., 1996; Butterworth, 2010). Given the importance of mathematical ability in contemporary life, a deficiency in this ability can be an impediment to effective function in Western society (Rivera-Batiz, 1992; Nelson et al., 2008).

Investigating basic numerical skills, and specifically numerical cognition, can be an efficient way to study the typical as well as atypical development of mathematical abilities. Indeed, a recent intriguing argument is that complex human cognitive achievements, such as mathematics and geometry, which are exclusively human in their linguistic and symbolic structure, rest on a set of evolutionarily early non-symbolic and approximate representations of quantities, with specialized cerebral subsystems (e.g., Dehaene, 1997; Spelke and Kinzler, 2007; Butterworth et al., 2008; Nieder and Dehaene, 2009). The link between basic cognitive skills and mathematics becomes evident in cases of atypical development of mathematical skills such as DD. Specifically, DD is reflected in deficits in several different basic numerical and functions such as spontaneous focusing on number (Hannula et al., 2010), comparing non-symbolic numerical quantities (e.g., Piazza et al., 2010; Halberda et al., 2012), processing numbers symbolically (e.g., in Arabic notation, Stock et al., 2010), or linking non-symbolic representations to symbols such as Arabic numerals (Bugden and Ansari, 2011; for review see Liane Kaufmann et al., 2013). Importantly, very recently, it has been shown that preschoolers (as early as ages 3–5 years old—Bonny and Lourenco, 2013; or even 6-months old infants Starr et al., 2013) who are shown precise number representations, are more mathematically competent than those with unstable number repersentations. Moreover, it had been suggested that an important part of the way to help people who suffer from DD is by strengthening basic numerical processing, for example, with a specially-designed program of activities (Butterworth and Laurillard, 2010) Similarly, training basic numerical skills such as non-verbal quantity manipulations, can enhance intact arithmetical skills (Park and Brannon, 2013, 2014; Hyde et al., 2014). Hence, it seems resonable to conclude that basic numerical skill and higher arithmetic and mathematical abilities and strongly connected.

Indeed, the field of numerical cognition has seen an upsurge of research in the last three decades. Such research has furnished the scientific community with knowledge on the foundations of numerical abilities and the brain mechanisms involved (Ansari and Karmiloff-Smith, 2002; Ansari, 2008). This research is relevant to educational practice and policy. So far, however, it has exerted little influence on mathematics education. This is due in part to the fact that it is not easy to apply the detailed findings from research in cognitive neuroscience with the different dynamic variables that take place in learning situations. For example, In the field of DD, findings and assessments are usually based on either explicit self-report questionnaires or explicit school-like tests such as the Woodcock Johnson (Woodcock, 1989, 1990; Woodcock et al., 2001) or the Wide Range Achievement (Wilkinson, 1993) tests. Because these explicit tests depend on intact higher-level cognitive processes, they may fail to provide detailed information regarding specific deficits in the more fundamental aspects of number processing. Explicit questionnaires and tests have been the primary method for obtaining information about learning disabilities and abilities in the school setting, in part for reasons of convenience, standardization, and good psychometric properties (as suggested for example in cases of ADHD—Pelham et al., 2005). Accordingly, the question of the neurocognitive source of DD is still under huge debate (e.g., two optional suggestions for the cognitive source are the core deficit hypothesis” Butterworth, 2010; vs. the “deficit in processing symbolic numerals” Mussolin et al., 2010b) despite its significance for education and daily life. Several different numerical dysfunctions are seen in DD, among which is the ability to compare non-symbolic numerical quantities (e.g., dot arrays: Piazza et al., 2010 ordinality: Rubinsten and Sury, 2011) and to process numbers symbolically (e.g., in Arabic notation—Stock et al., 2010; Furman and Rubinsten, 2012; for review see Kaufmann et al., 2013).

Taking individual differences and a cognitive neuroscientific framework as starting points, we review here two suggestions for optimizing the development of assessment tools: (1) use low level neurocognitive tests to assess the ability to manipulate very specific numerical information. Currently, learning disabilities in mathematics (or DD) cannot be diagnosed by biological markers. Instead, the diagnosis is based on behavioral criteria. However, DD and other learning disabilities such as Dyslexia are believed to be neuro-developmental disorders with a biological origin (e.g., Price et al., 2007; Soltész et al., 2007; Rotzer et al., 2008; Kaufmann et al., 2009; Kucian et al., 2011) and possess specific behavioral signs. These signs expand considerably beyond mathematical problems (which are behavioral symptoms) and relate, for example, to executive and linguistic dysfunctions. Hence, some causes of the behavioral signs, as well as symptoms of the condition, can, and maybe should be, diagnosed cognitively. (2) The potential utility of implicit learning paradigms for assessment of motivational constructs also warrants more attention. Neurocognitive tests can be designed to study not only intentional, but also non-intentional processing, that is, processing of information that is irrelevant to the task at hand. Research has shown that some deficiencies reveal themselves not when participants focus their attention upon the task rather only when the specific aspect is irrelevant to the task. Indeed, implicit measures can assess inaccessible cognitive structures or presentations that are being processed automatically.

In what follows we review examples of these two proposed guidelines and show how assessment can be made more efficient and precise when using them.

## Cognitive Neuroscience Methodologies as a Basis for Clinical Assessment

Features of mental or educational disorders are best understood in terms of deficits on the cognitive and biological levels (e.g., Frith, 2001). Even a single deficit on the behavioral or cognitive level may produce, through development, a cascade of difficulties (Rutter and Sroufe, 2000) that may eventually become comorbidity (e.g., math and reading difficulties) or appear to be a network of symptoms on the behavioral level (i.e., low achievements in school in several areas). As an example, the documentation of distinct neural substrates for different aspects of number processing (e.g., exact vs. approximate calculation; or linguistic representation of numerical symbols vs. magnitude representations; Nieder and Dehaene, 2009; Piazza, 2010; Santens et al., 2010) suggests that perturbations in one of the networks (either destruction, disconnection or malfunction) may give rise more than one cognitive deficit, for example, DD together with Dyslexia. Accordingly, evaluation of the network of core neurocognitive deficits may lead to a more accurate diagnosis of the more fundamental problem, which could enhance developments of specific intervention programs. Indeed, cognitive neuroscience research has identified cognitive and biological signatures that may serve to diagnose mental or educational impairments even earlier or more precisely than would be possible by behavioral inspection. Let us discuss these two features that are significant for the assessment of DD, i.e., early diagnosis and precise inspection, under the nexus of cognitive neuroscience and education. We bring the ability to process quantitative information as an example.

In the last three decades, it had been extensively argued that the cognitive foundation of mathematics rests on mental representations that developed in the course of evolution (Dehaene, 1992; Feigenson et al., 2004; Cantlon et al., 2009). These core representations include a non-symbolic numerical magnitude system that represents an approximation of the non-symbolic numerical value of a collection of objects (Dehaene, 2009).

Non-symbolic numerical knowledge is most commonly measured by comparison tasks, in which two arrays of dots are presented and the participant is asked to choose the larger one (Spelke, 2000; Holloway and Ansari, 2008; Piazza et al., 2010). One major feature of non-symbolic core numerical representations that is present in human adults, children, infants, and non-human animals is that speed and accuracy of processing are related to the ratio between the numbers being compared: accuracy falls and reaction time (RT) increases as the ratio of the numbers to be compared approaches one (i.e., the ratio effect, e.g., van Oeffelen and Vos, 1982; Dehaene, 1997; Barth et al., 2005, 2006). For example, Cantlon and Brannon (2006) trained monkey and human adults to discriminate two numerical stimuli based on their best estimate of the larger numerical value. For both groups accuracy and RTs were modulated by the numerical ratio between the stimuli. One corollary to this phenomenon is the distance effect; the larger the distance between two numbers to be compared the faster the response (i.e., the Distance effect, e.g., Ansari, 2008). This numerical distance effect was first reported by Moyer and Landauer (Moyer and Landauer, 1967) who suggested that people transform written or auditory numbers into analog magnitudes. Since their initial report, this effect has been reported by many other researchers under a variety of conditions (e.g., Dehaene, 1989; Schwarz and Stein, 1998; Holloway and Ansari, 2009; Kucian et al., 2011).

Further research has confirmed these effects (i.e., ratio and distance) in infants and animals, identified the brain tissue involved, and reported that these effects are compromised in developmental dyscalculia (DD). For example, the numerical distance effect has been found in children (Sekular and Mierkiewicz, 1997; Holloway and Ansari, 2009; Landerl and Kölle, 2009) and in primates (Nieder et al., 2002). Similarly, the ratio effect has been found in infants: (Xu and Spelke, 2000; Xu et al., 2005), in young children (Barth et al., 2005), and in animals: (Hauser et al., 2003; Cantlon and Brannon, 2007). Also, these two effects are compromised in DD (for the distance effect see Price et al., 2007; Soltész et al., 2007; Mussolin et al., 2010a; Heine et al., 2013; and for the ratio effect see Kovas et al., 2009; Libertus et al., 2011). Numerous studies have demonstrated that the parietal lobes and in particular the intraparietal sulcus (ips) plays a critical role in the mental operations involved in these effects (e.g., Dehaene et al., 2003; Fias et al., 2003; Piazza et al., 2004; Ansari and Dhital, 2006; Castelli et al., 2006; Cohen Kadosh et al., 2007).

This accumulated body of results has led to a widely accepted view of an innate domain-specific neural basis for numerical knowledge. Several investigators have suggested the existence of a core numerical system that facilitates the perception and manipulation of quantities (e.g., enumeration of dots; Nieder and Dehaene, 2009; Butterworth, 2010). Moreover, it has been suggested that DD (or mathematical learning disabilities) involves a domain specific deficit in the capacity to enumerate (Butterworth, 2010; Butterworth et al., 2011) as indicated for example, by different patterns (compared to controls who showed more linear patterns of the effects) of the ratio and distance effect (but see Kaufmann et al., 2013). Accordingly, it is very reasonable to use the ratio and distance effects as measures of math difficulties from a very young age.

To summarize, as with many other medical and educational conditions, early diagnosis can greatly facilitate remediation. As an example, Kroesbergen et al. (2012), showed that for kindergarten children who received training on either number sense or on both number sense and working memory, training number sense was the most effective. These findings indeed highlight the importance of early interventions for children at risk for mathematical learning problems. Indeed, as mentioned above, as early as preschool (e.g., 3–5 years old—Bonny and Lourenco, 2013; or 6-months old infants—Starr et al., 2013), children with efficient number sense, as indicated by precise number representations, are more mathematically competent than those with noisy number repersentations. In addition, Lonnemann et al. (2013), showed a significant link between the ratio effect and basic arithmetic in elementry school children. Hence, early diagnosis and intervention of very basic numerical skills can significantly enhance arithmetic abilities. However, early diagnosis poses several important challenges for clinicians and teachers such a poor stability of early diagnoses, limited usefulness of diagnostic tools for toddlers, and changes in patterns of symptoms in the first years of life. Quantitative analyses (i.e., analyzing numerical information in the environmental scene. e.g., numerical comparisons, numerical estimations), which have an important developmental interaction with numerical knowledge from infancy (e.g., Mix et al., 2002) and a clear neurocognitive signature (e.g., ratio and distance effects; Delazer and Butterworth, 1997; Turconi and Seron, 2002; Zorzi et al., 2011; Rubinsten et al., 2013), serve as important examples of the need for neurocognitive assessment tools. That is, humans’ evolutionarily and developmentally basic numerical abilities, including the internal representation of approximate numbers, provides a significant underpinning for the uniquely human mathematical skills, and may, therefore, provide the basis for new interventions for math educators (e.g., Park and Brannon, 2014). Accordingly, cognitive neuroscience may provide solutions for these challenges (i.e., limited stability of early diagnoses and usefulness of tools) since they offer knowledge of brain architectures that shape how we acquire math, language, reading, and more, and use detailed cognitive neuroscientific tools that enable early finding of cognitive deficits.

## Inspection of Non-Intentional Cognitive Variables as the Basis for Clinical Assessment

Implicit processes (Schacter, 1987; Reber, 1989) are viewed as part of a phylogenetically primitive system that expands to form conscious and explicit functioning during human development (Reber and Allen, 2009; such as learned arithmetic and mathematics). It has been argued and shown (e.g., Reber, 1989; Reber et al., 1991) that implicit knowledge in general is powerful and that it is important to assess implicit knowledge, particularly in cases of psychiatric or neurological deficiencies, which are typically assessed by the more traditionally explicit cognitive tests.

In the field of mathematics, several studies have found that when using implicit cognitive tasks [e.g., Continuous Flash Suppression (CFS)—Tsuchiya and Koch, 2005 a masking technique that enables subliminal presentations that last seconds], even the seemingly complex ability to solve arithmetic facts (e.g., single digit additions) is shown to have implicit and unaware aspects (Sklar et al., 2012; see also Rusconi et al., 2006; García-Orza et al., 2009; Ric and Muller, 2012). But what would be the methodological definition of implicit knowledge and, following that, what would be a good tool for assessing implicit knowledge? In implicit/explicit (or non-conscious/conscious) perception and knowledge, the role of attention has been the subject of substantial research (Merikle et al., 1995). While there are disagreements concerning the depth of processing of unattended stimuli, there is no doubt that attention serves as a filter preceding explicit perception or awareness (e.g., Driver and Vuilleumier, 2001). We bring here the example of the affective priming task as a tool for assessing the implicit neurocognitive constructs that underlie mathematical anxiety.

Math anxiety, a persistent negative reaction to math, ranges from mild discomfort to extreme avoidance (Hembree, 1990; Ma and Xu, 2004a, b; Ashcraft and Ridley, 2005; Maloney and Beilock, 2012). Specifically, math anxiety may include feelings of tension (Richardson and Suinn, 1972), low self confidence in the ability to learn mathematics (Jain, 2009), and poorer working memory (Ashcraft and Kirk, 2001), and counting abilities (Maloney et al., 2010), and a decline in the precision of the mental representations of numerical magnitudes (Maloney et al., 2011). Also, it had been shown that specific fronto-parietal cortical areas are involved with math anxiety (Lyons and Beilock, 2012; Young et al., 2012). Little is known however, about the determinants of sex differences in math anxiety (e.g., Betz, 1978; vs. Cooper and Robinson, 1991; or Hackett, 1985) and their reasons (for review see Maloney and Beilock, 2012) are still under huge debate. One variable that may influence reported sex differences in math anxiety is the common use of explicit tools such as the math anxiety rating scale (e.g., Richardson and Suinn, 1972), the math anxiety questionnaire (Wigfield and Meece, 1988; for a German version see: Krinzinger et al., 2007), or the revised Math Anxiety Rating Scale (MARS-R; Alexander and Martray, 1989; Hopko, 2003) to diagnose math anxiety. Such explicit tools typically assess accessible self-representations. Women, for example, have consistently been found to score higher than men on self-report measures of trait anxiety (e.g., Feingold, 1994; Costa et al., 2001; Egloff and Schmukle, 2004), due to gender differences in willingness to reveal personal or emotional information that is not necessarily related to anxiety *per se* (Flessati and Jamieson, 1991).

Implicit measures, on the other hand, typically assess inaccessible cognitive structures or presentations that are processed automatically. It has been shown that affective traits can be activated automatically and influence emotional, cognitive, or behavioral processes (e.g., Giner-Sorolla et al., 1999). That is, affective processing begins immediately and involuntarily upon seeing a salient affective word or picture. Egloff and Schmukle (2004) found that the effect sizes of gender differences in implicit anxiety measures were approximately half as large as those in the explicit tests. Such findings suggest that explicit anxiety measures may indeed be influenced (although not exclusively) by biased self-reports.

One cognitive tool for assessing implicit is the priming task, in which an early stimulus (i.e., prime; e.g., “yellow”), designed to be ignored, influences the response to a subsequent relevant stimulus (e.g., the target word “banana”). In many cases, participants cannot ignore the irrelevant dimension (the prime) because it is processed automatically without their direct attention. Hence, the irrelevant dimension facilitates or interferes with the processing of the relevant dimension (the target).

Accordingly, Rubinsten and colleagues (Rubinsten and Tannock, 2010), developed a novel arithmetic-affective priming task to study gender differences in math anxiety. Affective priming studies have demonstrated that people respond to target stimuli more quickly after presentation of an affectively related prime stimulus than after one that is affectively unrelated, whether the target involves written words or not (e.g., naming target’ written words: Hermans et al., 1994; Bargh et al., 1996; Cassotti et al., 2012; naming or categorizing pictures: Spruyt et al., 2004; facial recognition Suslow et al., 2013; for review see De Houwer et al., 2009).

The arithmetic-affective priming task (Rubinsten and Tannock, 2010) has four different types of affective primes. That is, primes could be words with positive (e.g., “love”), neutral (e.g., “table”), and negative (e.g., “war”) affect, as well as words related to mathematics such as “multiplication” or “quantity”. In addition, single-digit arithmetic problems (such as 3 + 4 = 7) acted as targets. Participants were required to decide if the target (i.e., the arithmetic problem) was true or false by pressing one of two optional keys on the keyboard. Using these primes and targets, it was found that affective priming indeed shows higher math anxiety levels in DD (Rubinsten and Tannock, 2010) and in females (Rubinsten et al., 2012).

Accordingly it might be argued that implicit measures and, more specifically, implicit math anxiety measures may provide an important tool when studying math differences.

## Conclusions

DD is a specific example of a neuro-developmental psychiatric disorders, one that is rooted in fundamental biological and cognitive processes but indicated only by behavioral signs. Therefore, even if at present research regarding such biological and cognitive deficits is not always conclusive, it can serve as a basis for testable predictions. Due to the clear and significant link between basic numerical cognition and later arithmetic skills, early diagnosis and intervention of numerical skills can significantly enhance arithmetic abilities.

In order to translate research conducted in cognitive neuroscience laboratories to education, and in order to facilitate early diagnosis of DD, it is important to work in tight collaboration with clinicians and educators. Such a collaborative work should be conducted while using the very important and relevant findings from cognitive neuroscience investigations, including the examination of non-intentional cognitive variables.

## Conflict of Interest Statement

The author declares that the research was conducted in the absence of any commercial or financial relationships that could be construed as a potential conflict of interest.
